# Prevalence and associated factors of osteoarthritis in the Ural Eye and Medical Study and the Ural Very Old Study

**DOI:** 10.1038/s41598-022-16925-6

**Published:** 2022-07-23

**Authors:** Mukharram M. Bikbov, Gyulli M. Kazakbaeva, Timur R. Gilmanshin, Rinat M. Zainullin, Ellina M. Rakhimova, Albina A. Fakhretdinova, Azaliia M. Tuliakova, Iuliia A. Rusakova, Songhomitra Panda-Jonas, Ildar F. Nuriev, Artur F. Zaynetdinov, Ainur A. Zinnatullin, Inga I. Arslangareeva, Ainur V. Gizzatov, Natalia I. Bolshakova, Kamilia R. Safiullina, Jost B. Jonas

**Affiliations:** 1 Ufa Eye Research Institute, 90 Pushkin Street, Ufa, Bashkortostan 450077 Russia; 2 Ufa Eye Institute, Ufa, Russia; 3 Privatpraxis Prof Jonas und Dr Panda-Jonas, Heidelberg, Germany; 4 Department of Ophthalmology, University Hospital Heidelberg, Heidelberg, Germany; 5 Department of Ophthalmology, Medical Faculty Mannheim, Heidelberg University, Theodor-Kutzerufer 1, 68167 Mannheim, Germany; 6 Institute of Molecular and Clinical Ophthalmology, Basel, Switzerland

**Keywords:** Osteoarthritis, Rheumatic diseases

## Abstract

To determine the prevalence of osteoarthritis and its associated factors in populations from Russia. The population-based Ural Eye and Medical Study (UEMS) and the population-based Ural Very Old Study (UVOS) were conducted in a rural and urban region in Bashkortostan/Russia and included participants aged 40+ and 85+ years, respectively. As part of a detailed systematic examination, we assessed the osteoarthritis prevalence in an interview including questions on the self-reported presence of osteoarthritis, the joints affected and osteoarthritis-related pain-relieving therapy taken. Out of 5898 participants of the UEMS, 1636 individuals had osteoarthritis [prevalence: 27.7%; 95% confidence interval (CI) 26.7, 28.7], with 816 individuals (13.8%; 95% CI 12.8, 14.8) taking pain-relieving medication. A higher osteoarthritis prevalence was associated (multivariable analysis) with older age [odds ratio (OR 1.04; 95% confidence interval (CI) 1.03, 1.05], urban region of residence (OR 1.25; 95% CI 1.07, 1.45), higher body mass index (BMI) (OR 1.04; 95% CI 1.03, 1.06), lower monthly income (OR 0.78; 95% CI 0.68, 0.90), higher physical activity score (OR 1.02, 95% CI 1.01, 1.03), higher prevalence of a history of cardiovascular disease including stroke (OR 1.55; 95% CI 1.33, 1.81), previous bone fractures (OR 1.20; 95% CI 1.04, 1.40) and previous falls (OR 1.22; 95% CI 1.03, 1.45), higher hearing loss score (OR 1.01; 95% CI 1.01, 1.02), and less alcohol consumption (OR 0.78; 95% CI 0.65, 0.93). Out of 1526 UVOS participants, 567 individuals had osteoarthritis (prevalence: 37.2%; 95% CI 35.0, 40.0), with 195 (12.8%; 95% CI 11.3, 14.3) individuals taking pain-relieving medication. Higher osteoarthritis prevalence was associated with rural region of habitation (OR 1.69; 95% CI 1.20, 2.38), lower monthly income (OR 0.62; 95% CI 0.46, 0.84), higher prevalence of cardiovascular disease (OR 1.75; 95% CI 1.30, 2.36), and higher anxiety score (OR 1.04; 95% CI 1.03, 1.06). Osteoarthritis and use of pain-relieving medication are common in these populations in Russia. Main associated factors were older age and lower monthly income in both study populations, female sex, higher BMI, urban region, and previous falls and bone fractures in the UEMS population, and rural region and a higher anxiety score in the UVOS study population.

## Introduction

Osteoarthritis (OA), as the most common form of arthritis in adults, results from a breakdown of joint cartilage and underlying bone^1–3^. It leads to symptoms such as joint pain and stiffness, joint swelling, and decreased range of motion. The knee and hip joints, joints of the neck and lower back, the middle and end joints of the fingers, and the metatarsophalangeal joint of the thumb are most commonly involved in patients with OA. Rarely occurring before the age of 40 years, the OA prevalence increases markedly with age. The public health-related importance of OA has been shown by its role as a major cause of health expenditure. Approximately 80 billion US$ were spent in relationship to OA in the USA in 2016^4^. As a corollary, OA has been described as one of the leading causes of disability in adults aged 60+ years in the 2015 World Health Organization (WHO) Global Ageing and Health Report^5^. In the Global Burden of Disease Study 2019, OA ranked at position #19 in the list of the leading causes of DALYs (disability-adjusted life years). It caused 1.5% of all DALYs in the age group of 50–74 years, and 1.1% of all DALYs in the age group of 75+ years^6^. Modifiable factors associated with OA include high body mass index (BMI), occupation, and history of injury, while non-modifiable factors for OA are parameters like older age and female sex^7–10^.

Despite its importance as a major cause of DALYs in the adult population, information about the prevalence of OA and its associated factors in Eastern Europe and Russia has remained scarce so far. Kalichman et al. examined in several studies the OA prevalence in subgroups such as in the Abkhazian, Chuvashian and Turkmen populations as well as in five Russian community-based samples^11–14^. Applying the Kellgren and Lawrence grading scheme and examining a group of 1005 Abkhazians with a mean age of 44.5 years, Kalichman and associates found an OA prevalence of the hand OA of 33.6% for men and 35.4% for women^11^. In participants aged > 65 years, the OA prevalence of the hand was 87.5% (men) and 83.3% (women), respectively. A higher OA prevalence was associated with a higher number of joints affected and higher BMI, while it was independent of sex. In a community-based group of 827 Chuvashians with a mean age of 49 years, the OA prevalence increased with older age, with a value of 89.2% in men and 97.6% in women in participants aged more than 65 years^12^. In a Turkmen community-based sample of 703 individuals aged 19-90 years, the OA prevalence increased from 13.8% in individuals aged less than 36 years to 100% in participants aged > 65 years^13^. In a sample of 1897 Russians with an age of 18–90 years, the OA prevalence was 98.5% in males and 96.8% in females, both aged > 65 years^14^. Using data from the World Health Organization Study on global ageing and adult health (SAGE), Brennan-Olsen and colleagues reported that the self-reported OA prevalence in a nationally-representative samples of older adults (≥ 50 years) in Russia was 38% (95% CI 36–39%) for men and 17% (95% CI 14–20%) for women^15^.

These preceding studies had limitations. They were performed in minority groups such as the Abkhazians and Chuvashians; the studies included only a small group of individuals aged 85+ years; and they usually did not examine a whole series of additional parameters and diseases which might have influenced the OA prevalence in a multivariable manner. We, therefore, evaluated the OA prevalence in two populations in Russia, with one population having a minimal age of 85 years, and we additionally examined a multitude of other factors and disorders to be included into a multivariable analysis. Knowledge about the OA prevalence as a major disease and its associated factors is essential to address the disorder and improve its prevention in public health measures.

## Methods

The individuals included in the present study were the participants of the Ural Eye and Medical Study (UEMS) and the Ural Very Old Study (UVOS). The UEMS is a population-based investigation which was performed in the Russian republic of Bashkortostan at the southwestern end of the Ural Mountains. The study lasted from 2015 to 2017^16,17^. Study regions were Ufa as the capital of Bashkortostan in a distance of about 1400 km East of Moscow, and a rural region in the Karmaskalinsky District in a distance of 65 km from Ufa. The republic of Bashkortostan, located between the Volga River and the Ural Mountains, has a population of 4 million people and is the most populous republic in Russia. The only inclusion criteria for the study were living in the study regions and an age of 40 years or older. There were no exclusion criteria. The Ethics Committee of the Academic Council of the Ufa Eye Research Institute approved the study design (protocol number 2, dated 25.8.2015) and confirmed that the study adhered to the Declaration of Helsinki. All participants gave an informed written consent. The study regions had been chosen due to their representativeness for a typical rural region in the countryside of Bashkortostan and for a typical urban area in the city of Ufa, respectively. Since all individuals aged 40+ years and living in the study regions were eligible for inclusion into the study, there were no sample designs such as a multi-stage sampling strategy or a proportional sampling scheme. Since the UEMS was designed to examine the prevalence and associated factors of a multitude of systemic and ophthalmological diseases and to assess the distribution of normative parameters such as blood pressure and body weight, we estimated the necessary size of the study population to be higher than 5000 participants. With an anticipated participation rate of 80%, the target population had to include more than 6000 individuals. As described in detail recently, out of a total group of 7328 eligible individuals, 5899 (80.5%) individuals (3319 [56.3%] women) with a mean age of 59.0 ± 10.7 years (range 40–94 years) participated in the UEMS^16,17^. The study population did not differ significantly in the gender and age distribution from the Russian population as explored in the census carried out in 2010^18^.

The UVOS is a population-based study, which was conducted in the same study regions as the UEMS, and which was performed in the period from 2017 to 2020^19^. The study was approved by the Ethics Committee of the Academic Council of the Ufa Eye Research Institute (protocol number 3, dated 10.8.2017) and informed written consent was obtained from all participants. Inclusion criteria were an age of 85+ years and living in the study regions. There were no exclusion criteria. Out of 1882 eligible inhabitants, 1526 (81.1%) persons participated in the study. The eligible individuals included the inhabitants of three private small retirement homes in the urban study region. There were no retirement homes in the rural study region. As already described recently, the participation rate did not vary markedly between the urban group [1238 (81.3%) out of 1523 individuals] and the rural group [288 (80.2%) out of 359 individuals]^19^. According to the census carried out in Russia in 2010, the composition of the population of the UVOS with respect to gender and age corresponded to the gender and age distribution in the Russian population beyond an age of 85+ years, with a marked preponderance of females^18^. With respect to the assessment of the study sample size and sampling strategies, the same facts described for the UEMS held true for the UVOS.

Using a bus, the study participants of both studies were brought from their homes to the Ufa Eye Institute where a team of about 20 trained technicians and ophthalmologists performed all examinations. Those individuals which were unable to come to the hospital, underwent the interview and all examinations, which could be performed outside of the hospital, in their homes. The OA presence was assessed in an interview including questions on the self-reported physician/clinical diagnosis of OA, the typical symptoms of OA, the joints affected by OA, and whether oral medication was taken to address OA-related pain. The interview additionally consisted of more than 250 other standardized questions on the socioeconomic background, including the self-reported ethnicity, level of education, occupation, family income and family estate (ownership of a house and second house, telephone, smartphone, laptop, television, bicycle and car), and size and structure of the family; diet (number of meals per day, frequency and amount of intake of vegetables, fruits, whole grain and meat, consumption of tea and coffee, use of animal fat or cooking oil); smoking (since when or stopped, cigarettes or other types of tobacco products, symptoms of smoking cessation); alcohol consumption (since when or stopped, alcohol consumption-related wrongdoing); physical activity (frequency and intensity of daily work, leisure time activities, sitting or reclining); quality of life and quality of vision; symptoms of chronic obstructive pulmonary disease, asthma, kidney disease and orthopedic disorders; history of any type of injuries and inter-personal violence; and health assessment questions^16,17,19^. The questionnaire additionally included questions on the medical history including known diagnosis and therapy of major disorders such as diabetes mellitus, arterial hypertension, cardiovascular diseases, headache, neck pain, thoracic spine and low back pain, depression, suicidal ideas, anxiety, questions on previous neurologic attacks including stroke, epilepsy, polyneuropathy and unconsciousness, and questions on cognitive function and hearing loss. The questions included in the interview were taken from standardized interviews published in the literature, such as the “Center for Epidemiologic Studies Depression Scale (CES-D) Scoresheet”, the Folstein test, Zung´s self-rated depression scale, the National Eye Institute Visual Functioning Questionnaire-25 (VFQ-25), the Questionnaire for Verifying Stroke-Free Status (QVSFS) from the American Heart Association, and the Michigan Neuropathy Screening Instrument^20–25^. The interview was conducted in the study participants’ homes when visited by the social workers or in the hospital.

The examinations further included anthropometry, blood pressure measurement, handgrip dynamometry, spirometry, and biochemical analysis of blood samples taken under fasting conditions. We defined arterial hypertension according to the criteria published by the American Heart Association. Criteria for the diagnosis of diabetes mellitus were a fasting glucose concentration of ≥ 7.0 mmol/L or a self-reported history of physician diagnosis of diabetes mellitus or a history of drug treatment for diabetes (insulin or oral hypoglycemic agents). Depression was assessed by applying the Center for Epidemiologic Studies Depression Scale (CES-D) Scoresheet^15^. The estimated glomerular filtration rate (eGFR) was calculated using the chronic kidney disease (CKD) Epidemiology Collaboration (CKD-EPI) equation^26^. We applied the Guidelines for Accurate and Transparent Health Estimates Reporting (GATHER statement guidelines)^27^.

Inclusion criteria for the present study were the availability of data on the self-reported OA prevalence. Using a statistical software package (SPSS for Windows, version 27.0, IBM-SPSS, Chicago, IL, USA), we assessed the main outcome parameter, i.e., the OA prevalence [expressed as mean and 95% confidence intervals (CI)], and searched for associations between the OA prevalence and other parameters, in univariable logistic regression analyses, followed by a multivariable logistic regression analysis. The latter included the OA prevalence as dependent variable and as independent parameters all those variables which were associated with the OA prevalence in the univariable analysis with a *P* value of ≤ 0.10. In a step-by-step manner, we dropped those parameters out of the list of independent parameters when they were no longer significantly associated with the OA prevalence. We then re-added parameters, which had previously dropped out, again to the model to re-test for the significance of their potential association with the OA prevalence. We calculated the odds ratio (OR) and the 95% CIs. All *P* values were two-sided and were considered statistically significant when the values were less than 0.05.

## Results

### Ural Eye and Medical Study

Out of 5899 participants of the Ural Eye and Medical Study, information about the presence of OA was available for 5898 (99.9%) individuals (2579 (43.7%) men) with a mean age 59.0 ± 10.7 years; range 40–94 years. The study participants included 1185 (20.1%) Russians, 1061 (18.0%) Bashkirs, 2439 (41.4%) Tatars, 587 (10.0%) Chuvash, 21 (0.4%) Mari, and 605 (10.3%) other or undefined ethnic groups.

OA was present in 1636 of the study participants with a prevalence of 27.7% (95% CI 26.7, 28.7) (Tables 1, 2). Pain-relieving tablets were taken by 816 individuals (13.8% (95% CI 12.8, 14.8) of the total population, or 49.9% of the group with arthritis). The OA prevalence was significantly higher in women than in men (*P* < 0.001), and it was higher in the urban region than in the rural region (*P* < 0.001) (Table 2).

**Table 1:** Prevalence of osteoarthritis in the Ural Eye and Medical Study and Ural Very Old Study, stratified by age and gender.

Age (years)	Men	Women	Total
**Ural Eye and Medical Study**			
40 to < 45	23/217 (10.6%; 95% CI 6, 15)	43/283 (15.2%; 95% CI 11, 19)	66/500 (13.2%; 95% CI 10, 16)
45 to < 50	43/357 (12.0%; 95% CI 9, 15)	80/387 (20.7%; 95% CI 17, 25)	123/744 (16.5%; 95% CI 14, 19)
50 to < 55	70/438 (16.0%; 95% CI 13, 19)	133/492 (27.0%; 95% CI 23, 31)	203/930 (21.8%; 95% CI 19, 24)
55 to < 60	98/489 (20.0%; 95% CI 16, 24)	187/550 (34.0%; 95% CI 30, 38	285 /1039 (27.4%; 95% CI 25, 30)
60 to < 65	95/405 (23.5%; 95% CI 19, 28)	208/516 (40.3%; 95% CI 36, 45)	303 /921 (32.9%; 95% CI 30, 36)
65 to < 70	64/292 (21.9%; 95% CI 17, 27)	222/499 (44.5%; 95% CI 40, 49)	286/791 (36.2%; 95% CI 33, 40)
70 to < 75	34/138 (24.6%; 95% CI 17, 32)	101/221 (45.7%; 95% CI 39, 52)	135/359 (37.6%; 95% CI 33, 43)
75 to < 80	44/165 (26.7%; 95% CI 20, 33)	116/247 (47.0; 95% CI 41, 53)	160/412 (38.8%; 95% CI 34, 44)
80+	22/78 (28.2%; 95% CI 18, 38)	53/124 (42.7%; 95% CI 34, 52)	75/202 (37.1%; 95% CI 30, 44)
Total	493/2579 (19.1%; 95% CI 18, 21)	1143/3319 (34.4%; 95% CI 33,36)	1636/5898 (27.7%; 95% CI 27, 29)
**Ural Very Old Study**			
85, 86	38/139 (27.3%; 95% CI 20, 35)	151/365 (41.4%; 95% CI 36, 46)	189/504 (37.5%; 95% CI 33, 42)
87, 88	36/101 (35.6%; 95% CI 26, 45)	112/300 (37.3%; 95% CI 32, 43)	148/401 (36.9%; 95% CI 32, 42)
89, 90	25/77 (32.5%; 95% CI 22, 43)	78/227 (34.4%; 95% CI 28, 41)	103/304 (33.9%; 95% CI 29, 39)
91, 92	11/40 (27.5%; 95% CI 13, 42)	61/144 (42.4%; 95% CI 34, 51)	72/184 (39.1%; 95% CI 32, 46)
93+	16/33 (48.5%; 95% CI 30, 66)	39/98 (39.8%; 95% CI 30, 50)	55/131 (42.0%; 95% CI 33, 51)
Total	126/390 (32.3%; 95% CI 28, 37)	441/1135 (38.9%; 95% CI 36, 42)	567/1525 (37.2%; 95% CI 35, 40)

**Table 2:** Demographic parameters of the population of the Ural Eye and Medical Study, stratified by the presence of osteoarthritis.

Parameter	Osteoarthritis present	Osteoarthritis absent	*P* value
**Ural Eye and Medical Study**			
n	1636	4262	
Age (years)	62.1 ± 10.2	57.8 ± 10.6	< 0.001
Men/women	493 (30.1%)/1143 (69.9%)	2086 (48.9%)/2176 (51.1%)	< 0.001
Rural/urban region of habitation	859 (52.5%)/777 (47.5%)	2541 (59.6%)/1721 (40.4%)	< 0.001
Family status: married/unmarried/divorced/widowed/missing	1116 (68.2%)/128 (7.8%)/100 (6.1%)/290 17.7%)	3198 (75.0%)/251 (5.9%)/238 (5.6%)/575 (13.5%)	< 0.001
Family status: married versus any other status	1116 (68.2%)/518 (31.7%)	3198 (75.0)/1064 (25.0%)	< 0.001
Family type: joint (three generations)/nuclear (two generations)/single/family of 2 people	367 (22.4%)/733 (44.8%)/106 (6.5%)/424 (25.9%	1176 (27.6%)/1773 (41.7%)/218 (5.1%)/1082 (25.4%)	0.02
Religion: muslim/christian/other	1009 (61.7%)/599 (36.6%)/28 (1.7%)	2661 (62.4%)/1525 (35.8%)/76 (1.8%)	0.65
Religion: muslim/any other religion	1009 (61.7%)/627 (38.3%)	2661 (62.4%)/1601 (37.6%)	0.59
Ethnicity: Russian/Bashkirs/Tatars/Chuvash/Mari/others/missing	334 (20.4%)/252 (17.9%)/673 (41.1%)/120 (7.3%)/2 (0.1%)/25 (1.5%)	851 (20.0%)/809 (19.0%)/1766 (41.4%)/467 (11.0%)/19 (0.4%)/79 (1.9%)	0.51
Ethnicity: Russian/any other ethnicity	334 (20.4%)/1072 (65.5%)	851 (20.0%) /3140 (73.7%)	0.06
Body height (cm)	162.6 ± 8.3	165.7 ± 8.8	< 0.001
Body weight (kg)	76.2 ± 14.9	75.7 ± 14.4	0.25
Body mass index (kg/m^2^)	28.8 ± 5.3	27.6 ± 4.8	< 0.001
Waist circumference (cm)	94.7 ± 13.9	93.7 ± 13.1	< 0.001
Hip circumference cm)	104.0 ± 13.8	103.5 ± 12.1	0.17
Waist/hip circumference ratio	0.91 ± 0.09	0.91 ± 0.09	0.02
Systolic blood pressure (mmHg)	134.9 ± 20.9	133.1 ± 20.2	0.002
Diastolic blood pressure (mmHg)	81.9 ± 10.6	82.0 ± 10.4	0.55
Mean blood pressure (mmHg)	99.5 ± 12.6	99.0 ± 12.5	0.18
Ankle-brachial index, right side	1.25 ± 0.19	1.25 ± 0.18	0.31
Ankle-brachial index, left side	1.25 ± 0.19	1.23 ± 0.18	0.09
**Socioeconomic parameters**			
Level of education	5.6 ± 1.4	5.6 ± 1.4	0.34
Monthly income (below poverty line/average/above average/high)	377 (23.0%)/1206 (73.7%)/52 (3.2%)/0	941 (22.1%)/3106 (72.9%)/206 (4.8%)/7 (0.2%)	0.04
Socioeconomic score	5.9 ± 1.5	5.9 ± 1.5	0.51
Physical activity score	7.4 ± 7.7	8.0 ± 8.2	0.008
**History of diseases**			
History of headache	582 (35,6%)/824 (50.4%)	2267 (53.2%)/1724 (40.5%)	< 0.001
History of neck pain	805 (49.2%)/601 (36.7%)	3022 (70.9%)/969 (22.7%)	< 0.001
History of thoracic spine pain	879 (53.7%)/527 (32.2%)	3247 (76.2%)/744 (17.5%)	< 0.001
History of back pain	428 (26.2%)/978 (59.8%)	2057 (48.3%)/1934 (45.4%)	< 0.001
History of therapy of hyperlipidemia	1157 (70.7%)/164 (10.0%)	3469 (81.4%)/272 (6.4%)	< 0.001
History of cancer	1569 (95.9%)/67 (4.1%)	4154 (97.5%)/108 (2.5%)	0.002
History of cardiovascular disorders including stroke	824 (50.4%)/582 (35.6%)	3104 (72.8%)/887 ( 20.8%)	< 0.001
History of dementia	1394 (85.2%)/12 (0.7%)	3966 ( 93.1%)/25 (0.6%)	0.38
History of diabetes mellitus	1420 (86.8%)/216 (13.2%)	3977 (93.3%)/284 (6.7%)	< 0.001
History of diarrhea	1392 (85.1%)/14 (0.9%)	3978 (93.3%)/13 (0.3%)	< 0.001
History of bone fracture	916 ( 56.0%)/490 (30.0%)	2831 (66.4%)/1160 (27.2%)	< 0.001
History of heart attack	1529 ( 93.5%)/107 (6.5%)	4054 (95.1%)/208 (4.9%)	0.01
History of iron-deficiency anemia	1282 (78.4%)/124 (7.6%)	3811 (89.4%)/180 (4.2%)	< 0.001
History of low blood pressure and hospital admittance	1528/93.4%)/102 (6.2%)	4128 (96.9%)/113 (2.7%)	< 0.001
History of skin disease	1304 (79.7%)/102 (6.2%)	3806 (89.3%)/24 (0.6%)	< 0.001
History of use of steroids	1625 (99.3%)/10 (0.6%)	4234 (99.3%)/24 (0.6%)	0.83
History of thyroid disease	1374 (84.0%)/262 (16.0%)	3913 (91.8%)/349 (8.2%)	< 0.001
History of falls	1235 (75.5%)/399 (24.4%)	3558 (83.5%)/702 (16.5%)	< 0.001
History of unconsciousness	1453 (88.8%)/183 (11.2%)	3955 (92.8%)/307 (7.2%)	< 0.001
Age of the last menstrual bleeding (years)	48.3 ± 5.1	48.3 ± 4.9	0.98
Age of last regular menstrual bleeding (years)	48.2 ± 5.1	48.1 ± 4.9	0.69
Menopause	87 (5.3%)/860 (52.6%)	476 (11.2%)/1492 (35.0%)	< 0.001
**Blood concentrations (mmol/L) of:**			
Alanine aminotransferase (IU/L)	21.8 ± 15,2	21.0 ± 10.6	0.02
Aspartate aminotransferase (IU/L)	21.1 ± 13.9	20.7 ± 9.7	0.23
Bilirubin, total (µmol/L)	14.2 ± 10.6	15.2 ± 11.4	0.005
High-density lipoproteins (mmol/L)	2.3 ± 0.9	2.3 ± 0.9	0.38
Low-density lipoproteins (mmol/L)	2.2 ± 2.3	2.1 ± 1.2	0.20
Triglycerides (mmol/L)	1.4 ± 0.8	1.4 ± 0.7	0.14
Cholesterol (mmol/L)	5.9 ± 2.0	5.8 ± 1.6	0.04
Erythrocyte sedimentation rate (mm/hour)	15.8 ± 11.9	13.6 ± 11.0	< 0.001
Glucose (mmol/L)	5.2 ± 1.8	5.0 ± 1.6	0.001
Creatinine (µmol/L)	87.6 ± 25.7	90.8 ± 24.6	< 0.001
Urea (mmol/L)	5.2 ± 1.5	5.1 ± 1.5	< 0.001
Residual nitrogen (g/L)	0.26 ± 0.05	0.25 ± 0.08	0.09
Total protein (g/L)	75.8 ± 6.2	76.0 ± 6.4	0.24
International normalized ratio (INR)	1.1 ± 0.1	1.1 ± 0.1	< 0.001
Blood clotting time (min)	3.8 ± 0.5	3.7 ± 0.5	0.06
Prothrombin index (%)	96.8 ± 10.0	95.7 ± 10.3	< 0.001
Hemoglobin (g/dL)	140.1 ± 14.7	143.2 ± 14.8	< 0.001
Erythrocytes (10^6^ cells/µL)	4.44 ± 0.37	4.50 ± 0.38	< 0.001
Leukocytes (10^9^ cells/L)	5.16 ± 1.45	5.11 ± 1.42	0.18
**Diet**			
Vegetarian diet/mixed diet	3 (0.2%)/1633 (99.8%)	7 (0.2%)/4255 (99.8%)	0.87
Number of meals per day	3.7 ± 0.8	3.6 ± 0.8	0.003
In a week how many days do you eat fruits?	5.2 ± 2.0	5.4 ± 2.0	0.59
In a week how many days do you eat vegetables?	6.2 ± 1.5	6.3 ± 1.4	0.12
Type of oil used for cooking: vegetable oil/non-vegetable oil	1138 (69.6%)/33 (2.0%)	15 (0.4%)/3326 (78.0%)	0.08
Food containing whole grains (yes/no)	289 (17.7%)/1117 (68.3%)	813 (19.1%)/3175 (74.5%)	0.89
Salt consumed per day (g)	4.3 ± 2.2	4.3 ± 2.4	0.54
Degree of processing of meat (weak/medium/well done)	26 (1.6%)/498 (30.4%)/880 (53.8%)	93 (2.2%)/1420 (33.3%)/2475 (58.1%)	0.51
**Miscellanea**			
Do you currently smoke any tobacco products? (yes)	1506 (92.1%)/127 (7.8%)	3641 (85.4%)/618 (14.5%)	< 0.001
Package years (package = 20 cigarettes)	3.0 ± 11.8	4.6 ± 13.0	< 0.001
Alcohol consumed such as beer, whisky, rum, gin brandy or other local products? (Yes/no)	1394 (85.2%)/242 (14.8%)	3245 (76.1%)/1015 (23.8%)	< 0.001
Hearing Loss Total Score	7.1 ± 12.7	4.4 ± 10.2	< 0.001
Depression score (adapted)	5.9 ± 3.8	4.9 ± 3.7	< 0.001
State-Trait Anxiety Inventory (STAI) score	7.0 ± 3.6	6.1 ± 3.5	< 0.001
Manual dynamometry, right hand (dekaNewton)	27.0 ± 10.7	31.8 ± 11.8	< 0.001
Manual dynamometry, left hand (dekaNewton)	23.7 ± 10.4	28.1 ± 11.4	< 0.001

In univariable analysis, a higher OA prevalence was associated with numerous variables including higher age, female sex, urban region of habitation, higher prevalence of widowed family status, lower body height, higher BMI, and higher hearing loss score (Table 2). Due to collinearity, we dropped in the multivariable analysis the variables of waist circumference, waist-hip circumference ratio, history of diabetes, and prevalence of current smoking. Due to a lack of statistical significance, we then dropped the parameters of body height (*P* = 0.79), marital status (*P* = 0.14), blood concentration of urea (*P* = 0.72), international normalized ratio (INR) (*P* = 0.77), serum concentration of aspartate aminotransferase (*P* = 0.56), glucose (*P* = 0.47) and bilirubin (*P* = 0.45), number of daily meals (*P* = 0.63), history of unconsciousness (*P* = 0.59), diarrhea (*P* = 0.43), cancer (*P* = 0.16), blood erythrocyte count (*P* = 0.22), number of smoking package years (*P* = 0.26), erythrocyte sedimentation speed (*P* = 0.15), history of heart attack (*P* = 0.13), blood concentration of hemoglobin (*P* = 0.17), depression score (*P* = 0.16), anxiety score (*P* = 0.49), prothrombin index (*P* = 0.10), serum concentration of alanine aminotransferase (*P* = 0.06), history of thyroid disease (*P* = 0.06), and dynamometric handgrip force (*P* = 0.06). In the final model, a higher OA prevalence was associated with various parameters such as older age, higher BMI, lower systolic blood pressure, lower monthly income, a higher score of physical activity, living in the city, a larger frequency of lower backache, cardiovascular disease, bone fractures, skin diseases, previous falls, lower serum concentration of creatinine, higher hearing loss score, and lower prevalence of any alcohol consumption (Table 3). The joints which were most often affected were the knee (70.1%), hip (9.6%), shoulder (4.2%), ankle (3.6%) and elbow (3.0%).

**Table 3:** Associations (multivariable analysis) between the prevalence of osteoarthritis and other parameters in the Ural Eye and Medical Study.

	Odds ratio	95% confidence interval		*P* value	Variance inflation factor VIF
		Lower value	Upper value		
Age (years)	1.04	1.03	1.05	< 0.001	1.39
Men/women	1.46	1.25	1.70	< 0.001	1.30
Rural/urban region of habitation	1.25	1.07	1.45	0.004	1.09
Body mass index (kg/m^2^)	1.04	1.03	1.06	< 0.001	1.15
Systolic blood pressure (mmHg)	0.995	0.992	0.999	0.015	1.23
Monthly income	0.78	0.68	0.90	0.001	1.02
Physical activity score	1.02	1.01	1.03	< 0.001	1.17
History lower backache	1.64	1.40	1.91	< 0.001	1.23
History of headache	1.21	1.05	1.40	0.009	1.17
History of neck pain	1.44	1.23	1.69	< 0.001	1.32
History of thoracic spine pain	1.52	1.29	1.79	< 0.001	1.30
History of cardiovascular disease including stroke	1.55	1.33	1.81	< 0.001	1.17
History of bone fracture	1.20	1.04	1.40	0.016	1.07
History of iron deficiency anemia	1.39	1.06	1.83	0.016	1.07
History of low blood pressure with hospital admission, falling or shock	1.56	1.12	2.18	0.009	1.03
History of skin diseases	1.34	1.01	1.78	0.043	1.02
History of falling	1.22	1.03	1.45	0.019	1.09
Serum concentration of creatinine (mmol/L)	0.996	0.993	0.999	0.015	1.09
Hearing loss score	1.010	1.005	1.02	0.001	1.10
Alcohol consumption, any	0.78	0.65	0.93	0.005	1.09

### Ural Very Old Study

Out of 1526 participants of the Ural Very Old Study, information about the presence of OA was available for 1525 (99.9%) individuals (390 (25.6%) men) with a mean age 88.3 ± 2.89 years; range 85–103 years. The study participants included 559 (36.7%) Russians, 171 (11.2%) Bashkirs, 669 (43.9%) Tartars, 49 (3.2%) Chuvash, 8 (0.5%) Mari, and 69 (4.5%) other or undefined ethnic groups.

OA was present in 567 of the study participants with a prevalence of 37.2% (95% CI 35.0, 40.0) (Tables 1, 4). Pain-relieving tablets were taken by 195 individuals (12.8% (95% CI 11.3, 14.3) of the total population, or 34.4% of the group with arthritis). The OA prevalence was significantly higher in women than in men (*P* = 0.02), and it was higher in the rural region than in the urban region (*P* < 0.001).

**Table 4:** Demographic parameters of the population of the Ural Very Old Study, stratified by the presence of osteoarthritis.

**Ural Very Old Study**			
n	567	958	
Age (years)	88.4 ± 3.0	88.3. ± 2.8	0.65
Men/women	126 (22.2%)/441 (77.8%)	264 (27.6%)/694 (72.4%)	0.02
Rural/urban region of habitation	142 (25.0%)/425 (75.0%)	146 (15.2%)/812 (84.8%)	< 0.001
Family status: married/unmarried/divorced/widowed/missing	113 (19.9%)/14 (2.5%)/7 (1.2%)/432 (76.2%)/1 (0.2%)	218 (22.8%)/12 (1.3%)/15 (1.6%)/704 (73.5%) 9 (0.9%)	0.29
Family status: married versus any other status	113 (19.9%)/453 (79.9%)	218 (22.8%)/731 (76.3%)	0.17
Family type: joint (three generations)/nuclear (two generations)/single/family of 2 people	103 (18.2%)/85 (15.0%)/193 (34.0%)/183 (32.3%)	155 (16.3%)/81 (8.5%)/361 /37.7%)/354 (37.0%)	0.88
Religion: Muslim/Christian/other	327 (57.7%)/232 (40.9%)/5 (0.9%)	519 (54.2%)/420 (4.8%) 12 (1.3%)	0.17
Religion: Muslim/any other religion	327 (57.7%)/237 (41.8%)	519 (54.2%)/432 (45.1%)	0.20
Ethnicity: Russian/Bashkirs/Tatars/Chuvash/Mari/others/missing	198 (34.9%)/66 (11.6%)/259 (45.7%)/16 (2.8%)/2 (0.4%)/25 (4.4%)	361 (37.7%)/105 (12.0%)/410 (42.8%)/33 (3.4%)/6 (0.6%)/36 (3.8%)	0.65
Ethnicity: Russian/any other ethnicity	198 (34.9%)/368 (64.9%)	361 (37.7%)/590 (61.6%)	0.25
Body height (cm)	158.2 ± 8.6	156.9 ± 9.4	0.04
Body weight (kg)	66.6 ± 11.4	65.2 ± 11.6	0.06
Body mass index (kg/m^2^)	26.7 ± 4.4	26.5 ± 4.5	0.51
Waist circumference (cm)	91.9 ± 11.5	92.0 ± 11.7	0.84
Hip circumference cm)	98.5 ± 11.2	98.4 ± 10.3	0.82
Waist/hip circumference ratio	0.93 ± 0.08	0.94 ± 0.09	0.71
Systolic blood pressure (mmHg)	157.9 ± 27.3	154.7 ± 25.1	0.30
Diastolic blood pressure (mmHg)	80.8 ± 15.2	78.7 ± 13.2	0.73
Mean blood pressure (mmHg)	106.5 ± 16.9	104.0 ± 15.2	0.72
Ankle-brachial index, right side	1.10 ± 0.06	1.10 ± 0.06	0.97
Ankle-brachial index, left side	1.10 ± 0.06	1.11 ± 0.06	0.65
**Socioeconomic parameters**			
Level of education	4.4 ± 2.1	4.6 ± 2.1	0.03
Monthly income (below poverty line/average/above average/high)	198 (34.9%)/355 (62.6%)/7 (1.2%)/2 (0.4%)	131 (13.7%)/795 (83.0%)/20 (2.1%) /1 (0.1%)	< 0.001
Socioeconomic score	10.1 ± 2.4	10.7 ± 2.3	< 0.001
**History of diseases**			
History of headache	228 (40.2%)/339 (59.8%)	583 (60.9%)/375 (39.1%)	0.24
History of neck pain	383 (67.5%)/184 (32.5%)	840 (87.7%)/118 (12.3%)	< 0.001
History of thoracic spine pain	344 (60.7%)/223 (39.3%)	724 (75.6%)/234 (24.4%)	< 0.001
History of back pain	144 (25.4%)/423 (74.6%)	485 (50.6%)/473 (49.4%)	< 0.001
History of therapy of hyperlipidemia	41 (7.2%)/520 (91.7%)	40 (4.2%)/910 (95.0)	0.054
History of cancer	522 (92.1%)/45 (7.9%)	883 (92.2%)/75 (7.8%)	0.78
History of cardiovascular disorders including stroke	133 (23.5%)/434 (76.5%)	418 (43.6%)/540 (56.4%)	< 0.001
History of dementia	508 (89.6%)/59 (10.4%)	901 (94.1%)/57 (5.9%)	0.66
History of diabetes mellitus	512 (90.3%)/55 (9.7%)	860 (89.8%)/98 (10.2%)	0.15
History of diarrhea	559 (98.6%)/8 (1.4%)	950 (99.2%)/8 (0.8%)	0.98
History of bone fracture	343 (60.5%)/224 (39.5%)	621 (84.8%)/337 (35.2%)	0.48
History of heart attack	472 (83.2%)/95 (16.%)	824 (86.0%)	0.001
History of iron-deficiency anemia	533 (94.0%)/34 (6.0%)	919 (95.9%)/39 (4.1%)	0.55
History of low blood pressure and hospital admittance	548 (96.6%)/18 (3.2%)	946 (98.7%)/5 (0.5%)	0.69
History of skin disease	524 (92.4%)/43 (7.6%)	910 (95.0%)/48 (5.0%)	0.33
History of use of steroids	562 (99.1%)/3 (0.5%)	924 (96.5%)/3 (0.3%)	0.04
History of thyroid disease	524 (92.4%)/43 (7.6%)	908 (94.8%)/50 (5.2%)	0.90
History of falls	256 (45.1%)/311 (54.9%)	489 (51.0%)/469 (49.0%)	0.03
History of unconsciousness	471 (83.1%)/96 (16.9%)	898 (93.7%)/60 (6.3%)	< 0.001
Age of the last menstrual bleeding (years)	51.0 ± 3.5	50.9 ± 3.9	< 0.001
Age of last regular menstrual bleeding (years)	49.4 ± 3.5	45.5 ± 3.9	0.03
**Blood concentrations (mmol/L) of**			
Alanine aminotransferase (IU/L)	17.2 ± 8.8	17.2 ± 8.8	0.13
Aspartate aminotransferase (IU/L)	26.3 ± 10.2	24.6 ± 10.0	0.13
Bilirubin, total (µmol/L)	13.7 ± 7.6	15.0 ± 9.4	0.02
High-density lipoproteins (mmol/L)	1.73 ± 0.80	1.74 ± 0.79	0.31
Low-density lipoproteins (mmol/L)	2.98 ± 1.07	2.95 ± 1.06	0.69
Triglycerides (mmol/L)	1.46 ± 0.73	1.45 ± 0.73	0.34
Cholesterol (mmol/L)	5.70 ± 1.20	5.61 ± 1.33	0.04
Erythrocyte sedimentation rate (mm/hour)	15.6 ± 12.9	14.6 ± 11.6	0.003
Glucose (mmol/L)	5.22 ± 1.54	5.12 ± 1.83	0.27
Creatinine (µmol/L)	101.3 ± 22.9	101.1 ± 22.8	0.14
Urea (mmol/L)	7.01 ± 2.38	6.92 ± 2.21	0.94
Residual nitrogen (g/L)	0.31 ± 0.07	0.31 ± 0.07	0.92
Total protein (g/L)	72.4 ± 7.6	71.2 ± 7.3	0.27
International normalized ratio (INR)	1.10 ± 0.12	1.10 ± 0.14	0.15
Blood clotting time (minutes)	3.78 ± 0.54	3.76 ± 0.53	0.43
Prothrombin index (%)	92.8 ± 9.2	92.6 ± 9.6	0.32
Hemoglobin (g/dL)	129.1 ± 17.7	128.8 ± 18	0.74
Erythrocytes (10^6^ cells/µL)	3.99 ± 0.51	3.97 ± 0.53	0.95
Leukocytes (10^9^ cells/L)	4.93 ± 1.70	4.94 ± 1.70	0.32
**Diet**			
Vegetarian diet/mixed diet	566 (99.8%)/1 (0.2%)	2 (0.2&)/944 (98.5%)	0.99
Number of meals per day	3.3 ± 0.8	3.53 ± 0.82	0.27
In a week how many days do you eat fruits?	4.81 ± 1.92	4.66 ± 2.07	0.76
In a week how many days do you eat vegetables?	6.32 ± 1.35	6.32 ± 1.35	0.01
Type of oil used for cooking: vegetable oil/non-vegetable oil	388 (68.4%)/17,410 (42.8%)0 (30.0%)	803 (83.8%)/138 (14.4%)	0.01
Food containing whole grains (no/yes)	37 (6.5%)/527 (92.9%)	50 (5.2%)/895 (93.4%)	0.56
Salt consumed per day (g)	4.42 ± 1.96	5.60 ± 1.88	< 0.001
Degree of processing of meat (weak/medium/well done)	15 (2.6%)/222 (39.2%)/319 (56.3%)	51 (5.3%)/479 (50.0%) /	< 0.001
**Miscellanea**			
Do you currently smoke any tobacco products? (no/yes)	560 (98.8%)/6 (1.1%)	953 (99.5)/5 (0.5%)	0.44
Alcohol consumed such as beer, whisky, rum, gin brandy or other local products? (No/Yes)	506 (89.2%)/61 (10.8%)	849/88.6%)/109 (11.4%)	0.01
Hearing Loss Total Score	17.9 ± 16.1	20.4 ± 16.9	0.04
Depression score (adapted)	10.3 ± 11.1	5.28 ± 8.21	< 0.001
State-Trait Anxiety Inventory (STAI) score	10.3 ± 11.1	− 0.8 ± 8.91	< 0.001
Manual dynamometry, right hand (dekaNewton)	12.8 ± 7.1	14.2 ± 7.7	0.22
Manual dynamometry, left hand (dekaNewton)	9.6 ± 6.6	11.2 ± 7.0	0.11

In univariable analysis, a higher OA prevalence was associated with various variables including female sex, lower educational level, rural region of habitation, higher hearing loss score, lower depression score, lower State-Trait Anxiety Inventory (STAI) score and others (Table 4). OA prevalence was not significantly (*P* = 0.65) associated with age. In the multivariable analysis we dropped due to a lack of statistical significance the parameters of socioeconomic score (*P* = 0.98), level of education (*P* = 0.35), history of thyroid disease (*P* = 0.89), fruit intake (*P* = 0.73), prevalence of any alcohol consumption (*P* = 0.52), history of falls (*P* = 0.69) and bone fractures (*P* = 0.90), serum concentration of cholesterol (*P* = 0.37), sex (*P* = 0.46), history of thoracic spine pain (*P* = 0.36), body height (*P* = 0.22), erythrocyte sedimentation rate (*P* = 0.44), depression score (*P* = 0.95), history of skin diseases (*P* = 0.31), serum bilirubin concentration (*P* = 0.11), and hearing loss score (*P* = 0.12). In the final model, a higher OA prevalence was associated with rural region of habitation, lower monthly income, higher prevalence of headache, neck pain and lower back pain, higher prevalence of a history of cardiovascular disease and unconsciousness, a higher number of days per week with vegetable intake and higher vegetable intake per day, higher salt consumption, higher degree of processing of meat, and higher anxiety score (Table 5). Joints which were most often affected were the knee (75.6%), hip (8.7%), hand (6.5%), shoulder (3.6%), and ankle (2.2%).

**Table 5:** Associations (multivariable analysis) between the prevalence of osteoarthritis and other parameters in the Ufa Very Old Study.

	Odds ratio	95% confidence interval		*P* value	Variance inflation factor VIF
		Lower value	Upper value		
Urban/rural region of residence	1.69	1.20	2.38	0.003	1.14
Monthly income	0.62	0.46	0.84	0.002	1.20
History lower backache	1.73	1.28	2.32	< 0.001	1.27
History of headache	1.61	1.22	2.11	0.001	1.15
History of neck pain	1.76	1.25	2.46	0.001	1.17
History of cardiovascular disease	1.75	1.30	2.36	< 0.001	1.18
History of unconsciousness	2.32	1.54	3.49	< 0.001	1.04
Number of days per week with vegetable intake	1.17	1.05	1.29	0.003	1.28
Amount of vegetable consumed per day of vegetable intake (g)	1.001	1.001	1.002	0.027	1.24
Salt consumed per day (g)	0.77	0.72	0.83	< 0.001	1.12
Degree of processing of meat	2.03	1.56	2.64	< 0.001	1.28
Anxiety score	1.04	1.03	1.06	< 0.001	1.24

## Discussion

In our middle-aged UEMS study population, the OA prevalence was 27.7%, with 816 individuals taking pain-relieving tablets. A higher OA prevalence was associated with factors such as older age, urban region of habitation, higher BMI, lower monthly income, higher physical activity score, higher prevalence of a history of cardiovascular disease including stroke and previous bone fractures, higher hearing loss score, and lower prevalence of any alcohol consumption. In the population of the UVOS, the OA prevalence was 37.2%, with 195 (12.8%) individuals taking pain-relieving tablets. A higher OA prevalence was associated with rural region of habitation, lower monthly income, higher prevalence of cardiovascular disease, and higher anxiety score.

The findings obtained in both of our studies agree with observations made in previous investigations conducted on study populations in other world regions^28–34^. In the United Kingdom-based Clinical Practice Research Datalink including data on more than 17 million patients, the prevalence of OA as diagnosed by general practitioners during the period from 1997 to 2017 was similar to the OA prevalence found in the UEMS population^28^. The OA prevalence was higher in women than in men and increased with older age. In a systematic review and meta-analysis on the prevalence of OA in Africa, Usenbo and colleagues reported that the OA prevalence was 55.1% in urban settings, and that it ranged between 29.5 and 82.7% in rural regions in South Africa among adults aged 65+ years^29^. In another meta-analysis, Yahaya and associates reported about a pooled OA prevalence of 16.1% in lower middle-income and low-income countries^30^. In a systematic analysis by the Global Burden of Disease Study 2017, Safiri et al. stated that the age-standardized prevalence of OA in 2017 was 3754 per 100,000 globally^31^. Women as compared to men had a higher OA prevalence which was additionally associated with older age. The OA prevalence was highest in the USA (6128/100,000), American Samoa (5281/100,000) and Kuwait (5235/100,000). In a cross-sectional population-based study conducted by Blanco and colleagues in Spain, the prevalence of symptomatic osteoarthritis was 29.4%, with the associated factors of female sex, low level of education and obesity^32^.

The OA prevalence found in our study populations concur with the results of previous investigations performed in Russia. Assessing OA according to the Kellgren and Lawrence grading scheme, Kalichman et al. reported about an OA prevalence of the hand of 33.6% for men and 35.4% for women in Abkhazians with a mean age of 44.5 years^11^. The OA prevalence increased in individuals aged > 65 years to 87.5% in men and 83.3% in women. The OA prevalence of 27.7% in the UEMS and of 37.2% in the UVOSs was lower than the figure of 89.2% in male Chuvashians and of 97.6% in female Chuvashians both aged > 65 years. They were also lower than the figures of 98.5% of male Russians and of 96.8% of female Russians aged > 65 years^12,14^. In the UVOS, the Chuvashians formed 587/5898 or 10.0% of the study population and had an OA prevalence of 20.4%. In the UVOS the Chuvashians formed 49/1525 or 3.2% of the study population and had an OA prevalence of 32.7%, with no significant difference in the OA prevalence as compared with the rest of the study populations. The figures of our study populations compare well with the data obtained from the SAGE study with a self-reported OA prevalence of 38% for men and of 17% for women in Russia with an age of ≥ 50 years^15^.

Factors associated with a higher OA prevalence were female sex in the UEMS and in the UVOS, a higher BMI and previous bone fractures in the UEMS. These findings agree with previous investigations in which these factors were associated with a higher OA prevalence^7–10,28–34^. The observations obtained in the UVOS are new since most previous studies were not focused on the very old population with age of 85+ years. Due to a minimal age of 85 years as inclusion criterion for the UVOS and the small age range of the UVOS population, the OA prevalence was not related to age in that study population. It may also be the reason for missing associations between the OA prevalence and other parameters such as BMI in the UVOS population as compared to the UEMS population.

In the UEMS, a higher OA prevalence was additionally associated with some blood biomarkers and parameters of lifestyle, such as a lower serum creatinine concentration and lower prevalence of alcohol consumption. In the UVOS, a higher OA prevalence was related to lower monthly income, higher intake of vegetables and more processed meat and lower salt intake (Tables 3, 5). The associations with lower monthly income may be due to a relationship between a higher OA prevalence and manual work versus desk-related work during lifetime, with manual work being associated with lower income. Correspondingly, a higher physical activity score was related to a higher OA prevalence in the UEMS population (Table 3). The relationship between higher OA prevalence and higher BMI, as also reported in previous studies, may be explained by the heavier body load on the main joints of knee and hip. In populations of the UEMS and the UVOS, history of cardiovascular disorders was correlated with a higher OA prevalence. While the cardiovascular system may not have a direct influence on arthritis, patients with arthritis may eventually avoid marked physical activity, what may lead to cardiovascular diseases. The association between a higher OA prevalence and a higher prevalence of previous falls or previous bone fractures may be explained by the traumatic joint changes caused by the falls. The reason of the association between a higher OA prevalence and a lower serum creatinine concentration has remained unclear; one may take into account the low statistical significance (*P* = 0.015) of this relationship in the multivariable analysis.

The prevalence of OA of 27.7% in the UEMS population and 37.2% in the UVOS shows the importance of OA for public health. Although one has to consider that there is no direct association between the prevalence of a disease and the years lived with disability due to the disease, our findings on the public importance of OA are paralleled by the results of the recent analyses of the Global Burden of Disease Study in which OA was the ninth-ranked cause of years lived with disability (YLDs)^35^. The finding of the knee joint as the most common site of OA in our study population corresponds with previous observations reported in the Global Burden of Disease Study^35^. It may be of particular concern that 13.8% of the UEMS population and 12.8% of the UVOS population took pain-relieving tablets. In view of the potential side effects of pain-relieving medications including chronic kidney disease, constipation, nausea, sedation, an increased risk of falls and fractures, depression, and sexual dysfunction, the large percentage of the study population taking pain-relieving medication for OA shows the importance OA in general and in particular with respect to the negative sequels of its symptomatic therapy^36,37^.

When results of our study are discussed, its limitations should be considered. First, the diagnosis of OA was not radiographically confirmed. However, it may not be feasible to perform an invasive X-ray examination for the participants of population-based studies. It may also be the reason for the global scarcity of population-based studies, in contrast to hospital-based investigations, on the OA prevalence. Second, differences between investigations in the composition of the study populations with respect to factors associated with the OA prevalence should be considered when the results of different studies are compared. Third, the validity of the method employed to identify individuals with OA may be a major point of concern. The diagnosis of OA is usually based on a clinical examination despite the widespread use of imaging methods^38–40^. The diagnosis of OA is often difficult even in a clinical and hospital setting, since there are no firmly established radiological criteria, due to a poor correlation between radiological findings and clinical functional impairments and pain^38–40^. In a similar manner, the diagnosis of OA based on an interview, albeit often applied in primary care situations as well as in our study, cannot use well established tools, since these have not been developed yet. In our study, we used questions about the physician-based or clinical diagnosis of OA and about the presence of typical OA-related symptoms, which joints were affected, and whether oral pain-relieving medication was taken. As a weakness in our study design, we did not test the validity of this approach, and whether the reported symptoms might not have been due to other diseases such as rheumatoid arthritis. Fourth, although the participation rates in the UEMS and OVOS were relatively high, the results of any population-based study have to be discussed with respect to their generalizability. The latter is based in particular on the representativeness of the study population. In both our studies, a major bias in the inclusion of study participants might have been unlikely. The study regions, a major city and a rural region in the Southern Russian republic of Bashkortostan southwest of the Ural Mountains was typical for the whole region of Southern Russia. Despite its relatively southern location, its continental climate with cold, harsh and long winters and warm to hot summers is comparable with the continental climate in North-Western Russia and Central Russia. The multi-ethnic composition of our study population was typical for Southern Russia and showed as compared to North-Western Russia and Central Russia a lower percentage of Russians on the total population. To address this potential limitation, we examined the OA prevalence in relationship to the ethnic background and did not find any correlation between both parameters, neither in the univariable analyses nor in the multivariable analyses. The age and sex distribution in our study populations was comparable to the results of the latest Russian census performed in 2010^18^. Fifth, the OA prevalence was not related to age in the UVOS, since the UVOS study population was selected on age and the age range of the study population was relatively small. Strengths of our project are that the OA prevalence has not been assessed yet in Eastern Europe and Russia, and that the UVOS is one of the very few studies worldwide including a very old population with an age of 85+ years.

In conclusion, OA and use of pain-relieving medication are common in these populations in Russia, with the main associated factors of older age and lower monthly income in both study populations, female sex, higher BMI, urban region, previous falls and bone fractures in the UEMS population, and rural region and higher anxiety score in the UVOS study population. Due to the age-dependence of the OA prevalence, the growing ageing epidemic may further increase the importance of OA for global public health.

## Data Availability

All identified data are available upon reasonable request from the corresponding author.
